# Meeting international self-report muscle strengthening guidelines is associated with better cardiovagal baroreflex sensitivity in adults

**DOI:** 10.3389/fspor.2024.1509784

**Published:** 2024-12-11

**Authors:** Jocelyn Waghorn, Beverly D. Schwartz, Madeline E. Shivgulam, Yanlin Wu, Derek S. Kimmerly, Myles W. O’Brien

**Affiliations:** ^1^ Division of Kinesiology, School of Health and Human Performance, Faculty of Health, Dalhousie University, Halifax, NS, Canada; ^2^ Geriatric Medicine Research, Dalhousie University & Nova Scotia Health, Halifax, NS, Canada; ^3^ Department of Medicine, Université de Sherbrooke, Sherbrooke, QC, Canada; ^4^ Centre de Formation Médicale du Nouveau-Brunswick, Université de Sherbrooke, Moncton, NB, Canada

**Keywords:** autonomic function, resistance training, physical activity guidelines, blood pressure regulation, cardiovagal baroreceptor sensitivity

## Abstract

Engaging in muscle strengthening activities (e.g., resistance training) at least twice/week is promoted by (Inter)national movement guidelines. Cardiovagal baroreflex sensitivity (cvBRS) reflects the ability to modulate R-R interval in response to changes in systolic blood pressure. Given the current conflicting literature, this study posed to explore the relationship between self-report muscle strengthening frequency and spontaneous cvBRS. 114 adults (62 females; age: 33 ± 19 years, 22% >55 years; body mass index: 24.2 ± 3.7 kg/m^2^) self-reported their weekly muscle strengthening activity frequencies via the Physical Activity and Sedentary Behaviour Questionnaire. Supine beat-by-beat R-R intervals [electrocardiography; 1.00 ± 0.18 s (0.90–1.50 s)] and systolic blood pressure [via finger photoplethysmography; 116 ± 11 mmHg (93–151 mmHg)] were recorded for 10.7 ± 2.0 min (5.3–14.5 min). Spontaneous cvBRS was assessed using the sequence technique. Data were analyzed using multiple regressions adjusted for age, sex, body mass index. Participants completed 2 ± 2 (0–7) days/week of muscle strengthening activities (56% met guidelines), and average overall cvBRS was 14.9 ± 9.1 (3.1–48.4) ms/mmHg. Higher reported frequencies were positively associated with overall cvBRS (Adjusted R^2^ = 0.40, *p* < 0.001; *β* = 2.24, *p* < 0.001). Meeting muscle strengthening activity guidelines was associated with improved overall cvBRS (Adjusted R^2^ = 0.29, *p* < 0.001; *β* = 7.68, *p* < 0.001). All results were unchanged if cvBRS for up-sequences or down-sequences only were used (all, *p* < 0.001). In conclusion, engaging in muscle strengthening exercises and particularly meeting existing guidelines were associated with better beat-by-beat vagally-mediated blood pressure regulation.

## Introduction

Muscle strengthening activities, including resistance training, are encouraged as a beneficial form of exercise that builds and maintains muscle mass (1), improves body composition (2), strengthens bone density (3), increases cognitive function (4), and lowers blood pressure (5, 6). The Canadian 24-h Movement Guidelines for adults (7) and World Health Organization (8) recommend engaging in muscle strengthening activities (e.g., resistance training, very heavy gardening, etc.) at least 2 times per week. Compared to aerobic exercise, there is limited research on the cardiovascular impacts of resistance training, with early work contributing to the dogma that it may promote arterial stiffness (9, 10). However, recent scientific statements made by the American Heart Association concluded that resistance training can lead to improvements in both traditional (e.g., blood pressure, glycemia, and body composition) and non-traditional (e.g., arterial stiffness, endothelial function, cardiorespiratory fitness) risk factors for cardiovascular disease (11). The American Heart Association (11) reported that resistance and aerobic training improve blood pressure to a similar degree (12), and that adding resistance training in combination with aerobic exercise may provide more benefits to reducing the risk of cardiovascular disease than aerobic training alone. It is important to consider the impact that resistance training has on specific markers of cardiovascular health, including arterial blood pressure (13). A meta-analysis demonstrated resistance training effectively lowered blood pressure in healthy adults (6). However, the magnitude of improvement was dependent upon the mode of resistance training, with greater reductions in blood pressure resulting from isometric handgrip exercises when compared to dynamic resistance training (6). Overall, additional evidence to support or refute the cardiovascular impacts of muscle strengthening exercises are needed.

Blood pressure is regulated on a beat-by-beat basis by the autonomic nervous system whereby the arterial baroreflex modulates sympathetic and cardiovagal activity to maintain blood pressure around an operating point. Cardiovagal baroreflex sensitivity (cvBRS) refers to the sensitivity of the baroreflex in modulating R-R interval (RRI) caused by changes in systolic blood pressure (SBP) (14, 15). Poor cvBRS is associated with a greater risk of hypertension and the occurrence of a major negative cardiovascular event (16). Understanding the impact of the frequency of weekly muscle strengthening exercise on cvBRS may provide important information on the cardiovascular benefits of resistance training.

Although there are complexities in the relationship between resistance training and blood pressure regulation (17, 18), there appears to be a consensus on the favorable impact of resistance training on blood pressure (6). However, current literature has mixed evidence as to whether it improves (19), does not change (20, 21), or decreases cvBRS (22). Specifically, low intensity (30% 1-repetition-maximum), low frequency (maximum 3 days/week) isometric handgrip exercise training for 8 weeks improved cvBRS in treated hypertensives (19). Lower cvBRS has been observed in males who strength trained 5 days/weeks for >2 years compared to untrained controls (22). In contrary, cvBRS was unchanged follow 8 weeks of 3 days/week of heavy resistance training in young adults (20), and 10 weeks of 3 days/week of periodized total body resistance training also in young adults (21). Given the contradicting results in the field, the primary purpose of this study was to (1) determine the relationship between muscle strengthening activity frequency and cvBRS, and (2) assess the impact of meeting the muscle strengthening activities guidelines (i.e., 2 days/week) on cvBRS. Given the conflicting reports (19, 20, 22), we did not have a directional hypothesis; however, with the support of public health activity guidelines, we believe that higher frequency habitual muscle strengthening will be associated with higher cvBRS.

## Methods

### Participants

One-hundred fourteen healthy adults (33 ± 19 years, 62 females) were included in this study. A sub-sample of the cvBRS outcomes (79/114), but not the frequency of muscle strengthening sessions, have been previously presented (23). However, this study answered an independent, novel research question on the impact of muscle strengthening activity frequency on cvBRS. Individuals were not excluded based on blood pressure or body mass index (BMI) cut-offs. Based on a medium effect size (f^2^ = 0.15), a multiple linear regression model indicated that a minimum of 92 participants were needed for five independent variables [muscle strengthening activity, moderate-vigorous aerobic physical activity (MVPA), age, sex, BMI] assuming a two-tailed, *α* = 0.05 and *β* = 80% power [G*Power, v3.1 (24)]. Three participants had a resting SBP >140 mmHg, and 2 participants had resting diastolic blood pressure (DBP) >90 mmHg. No participants were taking medication for high blood pressure. Of the 62 females, 46 were pre-menopausal and using oral contraceptive (*n* = 22), an intrauterine device (*n* = 5), Nexplanon (*n* = 1), or naturally menstruating (*n* = 18). Twenty of the pre-menopausal females were tested during the low estrogen phase (*n* = 12 using oral contraceptives), but menstrual phase was uncontrolled in the remaining 26. All protocols and procedures followed the Declaration of Helsinki, except for registration in a database, and were approved by the Dalhousie University Health Sciences Research Ethics Board. During the initial visit, the methods and experimental design were explained to the participants verbally and in writing before written, informed consent was provided.

### Experimental protocol

#### Anthropometrics and muscle strengthening activity frequency

Prior to the measurement session, participants were asked to refrain from consuming foods and supplements known to have acute effects on vascular function (e.g., caffeine, chocolate, saturated fats, folic acid supplements, antioxidant supplements, multivitamins) for >12-h (25). Participants were asked to abstain from moderate-to-vigorous activity >24-h prior to the laboratory session, alcohol consumption (>12-h) prior to testing (25). Height (to the nearest 0.1 cm) and weight (to the nearest 0.1 kg) were measured using a calibrated stadiometer (Health-O-Meter, McCook Il, USA). BMI was calculated as body mass (kg)/stature^2^ (m^2^).

The frequency of muscle strengthening sessions and self-reported MVPA were derived using the Physical Activity and Sedentary Behaviour Questionnaire (26). Participants answered the question, “How many days per week do you typically partake in muscle strengthening activities such as resistance training or very heavy gardening?”. The questionnaire is included in the Canadian Society for Exercise Physiology – Physical Activity Training for Health manual and was filled out on paper independently by each participant. Further, the participants were categorized by meeting, or not meeting the muscle strengthening activity guidelines cut-off of at least two days per week (7). MVPA is determined in this questionnaire via a physical activity vital sign by multiplying their answers to, “In a typical week, how many days do/did you do moderate-intensity (like brisk walking) to vigorous-intensity (like running) aerobic physical activity?” by “On average for days that you do/did at least moderate-intensity aerobic physical activity (as specified just above), how many minutes do/did you do?” (26).

#### Systemic hemodynamics

Beat-by-beat SBP and DBP were collected using finger photoplethysmography (Portapres®; Finapres Medical Systems, Amsterdam, the Netherlands), at a sampling rate of 200 Hz. Seated brachial SBP and DBP and supine brachial SBP and DBP were measured in triplicate using an automated vital signs monitor (Carescape v100; General Electric Healthcare, Mississauga, ON, Canada) to confirm eligibility and to calibrate the Portapres® signal, respectively. Heart rate (HR) and RRI were measured from the cardiac intervals derived from lead II of a standard bipolar limb lead electrocardiogram sampled at 1,000 Hz. All data were averaged over ∼10-min [average duration: 10.7 ± 2.0 min (5.3–24.5 min)] following a previous ≥15-min resting period of supine rest. LabChart software (Version 8.1.25; ADInstruments, Sydney, Australia) was used to view the recorded signals from a PowerLab data acquisition system (PL3508 PowerLab 8/53; ADInstruments, Sydney, Australia) in real-time and offline for analysis. Resting beat-by-beat SBP and RRI data collected in LabChart were exported as a.txt file to CardioSeries (V2.4, Brazil) for further analysis.

#### Spontaneous cardiovagal baroreflex sensitivity

Spontaneous cvBRS was calculated from resting SBP and RRI. The sequence technique, a common non-invasive method of measuring changes in RRI in relation to corresponding changes in SBP (27), was used and all cvBRS sequences were analyzed via CardioSeries software (V2.4, Brazil). CardioSeries (V2.4, Brazil) detects when there have been simultaneous and progressive increases or decreases in both SBP and RRI, referred to as up-sequences and down-sequences, respectively (28). The thresholds to be included as a sequence were ≥1 mmHg beat-by-beat changes in SBP and ≥1 ms changes in RRI (29). A minimum of 3 sequences were required to be included. There was a linear regression cut-off of *r* ≥ 0.85 when associating SBP and RRI for each up- and down-sequence (29). Overall, cvBRS was measured using the average slope of SBP and RRI regressions for the pooled sequences, as well as separately for the up- and down-sequences. Baroreflex effectiveness index (BEI) was measured as the ratio of the number of SBP ramp-induced changes in RRI to the total number of SBP ramps observed. The BEI was also reported separately for up- (up-BEI) and down-sequences (down-BEI).

#### Statistical analysis

Multiple regressions were used to evaluate the relationship between muscle strengthening exercise frequency per week (scored: 0–7) or meeting guidelines (Yes = 1, No = 0) with both cvBRS and BEI. This analysis was conducted on overall cvBRS and BEI, as well as for up-sequences and down-sequences only, average SBP and DBP, and average RRI. All regressions were covariate-adjusted for MVPA, age, sex, and BMI given the heterogeneous characteristics of the participants included. Multicollinearity was assessed via variance inflation factors, which were less than the standard threshold of 10 (all, <2.2) (30). To examine which of the independent variables were most strongly related to cvBRS, we conducted relative importance analysis in conjunction with regression analysis (31). This allowed the estimation of the raw weight that each variable contributes to the overall model. The statistical significance of the weights was determined via 10 000 replication bootstrapping, with statistical significance denoted by 95% confidence intervals not encompassing zero. All statistics were completed in SPSS Version 28.0.1.1 (14) (IBM, NY). Statistical significance was accepted as *p* < 0.05. All data are presented as means ± standard deviations (ranges) or proportions (%).

## Results

Detailed participant characteristics are presented in Table 1. Average overall cvBRS was 14.9 ± 9.1 ms/mmHg (3.1–48.4 ms/mmHg) and average BEI was 0.48 ± 0.15 (0.05–0.94).

**Table 1:** Descriptive, and hemodynamic outcomes for the pooled sample.

Variable	Participant characteristics
Descriptive	
Sex (M = 0; F = 1)	62 females, 52 males
Age (years)	33 ± 19 (19–83)
Participants >55 years	25 (22%)
Males	9/25 (36%)
Females	16/25 (64%)
Height (m)	170.5 ± 8.1 (145.5–190.0)
Weight (kg)	70.7 ± 12.9 (41.0–108.0)
BMI (kg/m^2^)	24.2 ± 3.7 (17.1–36.7)
Participants >30 kg/m^2^	6 (5%)
MVPA (mins/week)	240 ± 202 (15–1,680)
Muscle strengthening frequency	
0 days/week	28/114 (25%)
1 day/week	22/114 (19%)
2 days/week	14/114 (12%)
3 days/week	13/114 (11%)
4 days/week	16/114 (14%)
5 days/week	8/114 (7%)
6 days/week	8/114 (7%)
7 days/week	5/114 (4%)
Hemodynamic	
SBP (mmHg)	116 ± 11 (93–151)
Participants >140 mmHg	3 (3%)
DBP (mmHg)	64 ± 11 (43–116)
Participants >90 mmHg	2 (2%)
HR (beats/min)	66 ± 9 (47–90)
RRI (s)	1.00 ± 0.19 (0.67–1.23)
Cardiovagal function	
Up cvBRS (ms/mmHg)	13.8 ± 11.1 (1.9–63.0)
Down cvBRS (ms/mmHg)	14.8 ± 9.1 (0.5–44.4)
Up BEI (ratio)	0.37 ± 0.17 (0.05–0.88)
Down BEI (ratio)	0.60 ± 0.20 (0.04–0.97)
Up sequences (# of)	17.6 ± 14.8 (1.0–73.0)
Down sequences (# of)	30.5 ± 24.4 (1.0–117.0)

Data presented as means ± SD (range) or frequencies (%). BMI, body mass index; MVPA, moderate to vigorous physical activity; HR, heart rate; SBP, systolic blood pressure; DBP, diastolic blood pressure; MAP, mean arterial pressure; RRI, R-R interval; BEI, baroreflex efficiency index; cvBRS, cardiovagal baroreflex sensitivity.

**Figure 1:**
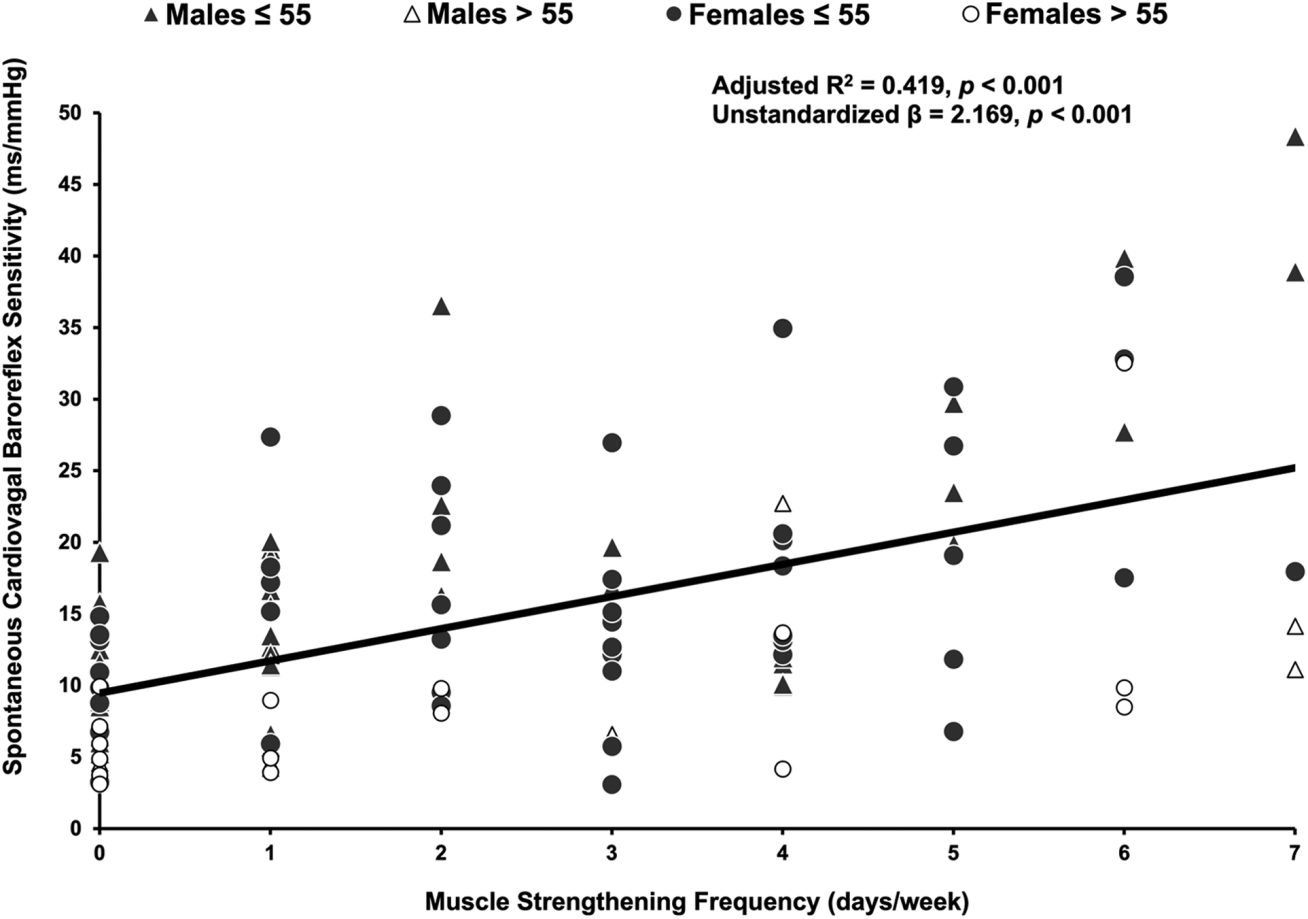
Multiple regression of muscle strengthening frequency and spontaneously measured cardiovagal baroreflex sensitivity. Participants (*n* = 114, 62 females) are grouped by age and sex, with triangles representing males, circles representing females, grey representing those 55 years or younger, and white representing those over 55 years. The multiple regression included moderate-to-vigorous physical activity, age, sex, and body mass index as covariates.

**Figure 2:**
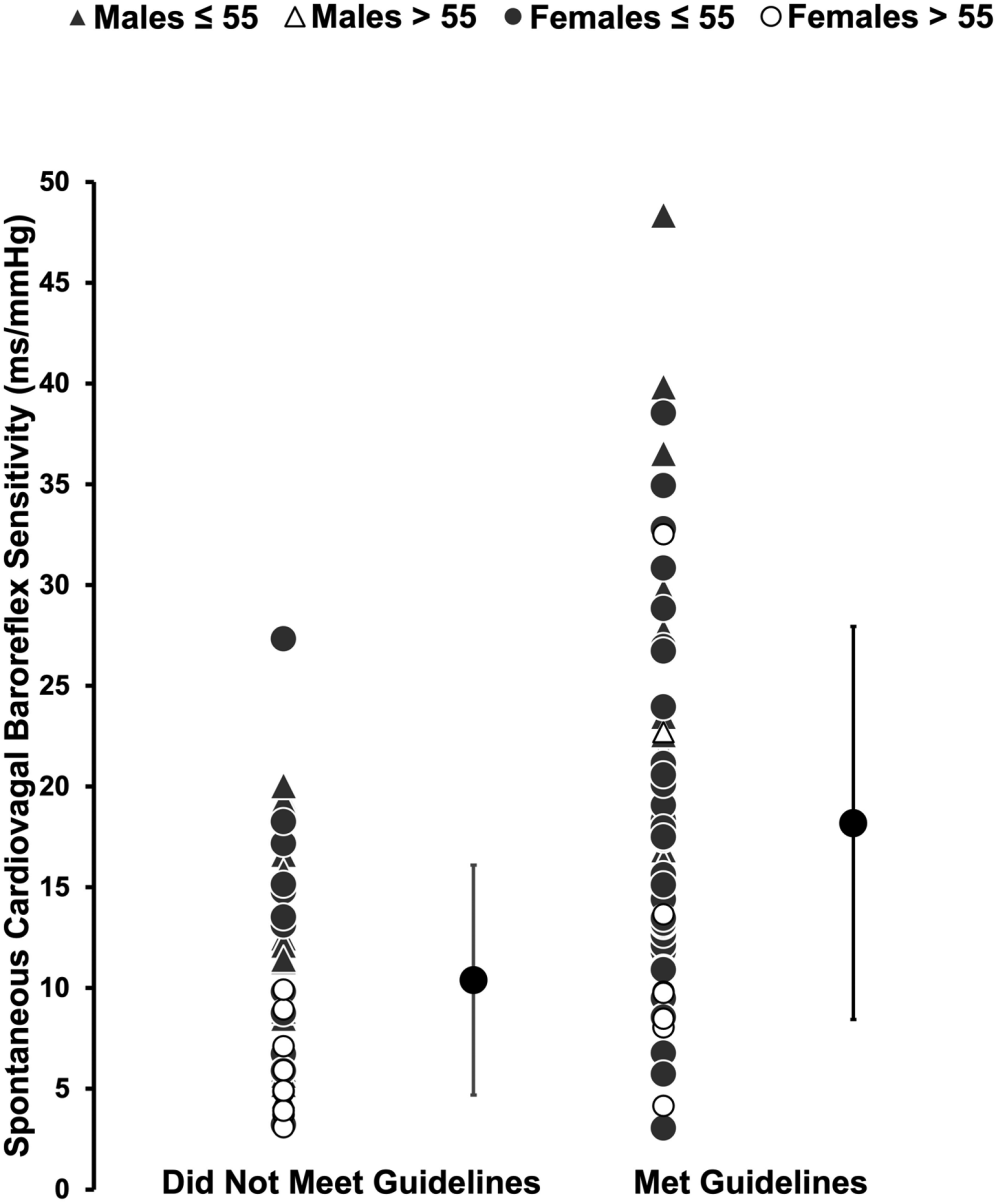
Comparing the spontaneously measured cardiovagal baroreflex sensitivity of those who met muscle strengthening guidelines to those who did not. Participants (*n* = 114, 62 females) are grouped by sex and age with triangles representing males, circles representing females, grey representing those 55 years or younger, and white representing those over 55 years. Mean and standard deviation of each group, those that did not meet guidelines and those that did are represented by the large black circles. The multiple regression included moderate-to-vigorous physical activity, age, sex, and body mass index as covariates.

Muscle strengthening activity frequency was positively correlated with overall cvBRS in the covariate-adjusted model (overall model: adjusted R^2^ = 0.419, *β* = 2.17, both, *p* < 0.001; raw weight 0.27; Figure 1, Table 2), as well as with up- and down-sequences (Supplementary Figures S1, S2). A sensitivity analysis removing those who engaged in 0 days/week did not change any results. Relative weights analysis confirmed that muscle strengthening activity frequency was the primary predictor for overall-, up-, and down- cvBRS (range: 50%–68%), with age being the only other significant predictor of overall- and down- cvBRS (range: 26%–38%) (Table 2). Meeting muscle strengthening frequency guidelines was also associated with a greater overall cvBRS (overall model: adjusted R^2^ = 0.306, *β* = 7.23, both, *p* < 0.001; raw weight 0.17; Figure 2; Supplementary Table S2), and up- and down-sequences (Supplementary Figures S3, S4). Relative weights analysis confirmed that meeting muscle strengthening guidelines was the primary predictor for overall-, up- cvBRS, and a secondary predictor for down- cvBRS (range: 41%–55%), with age being the only other significant predictor for overall- and down- cvBRS (range: 33%–46%) (Supplementary Table S2).

**Table 2:** Multiple regression analyses examining cardiovagal baroreflex sensitivity measures and their relation to muscle strengthening frequencies.

Variable	Unstandardized *β* (95% CI)	SE	*t*-value	Significant predictor (*p-*value)	Relative weight (% of 100%)
Overall cvBRS					
Muscle Strengthening Frequency (days/week)	2.169 (1.556, 2.782)	0.309	7.013	YES (<0.001)	59.74^a^
Age (years)	−0.159 (−0.234, −0.085)	0.038	−4.233	YES (<0.001)	26.26^a^
BMI (kg/m^2^)	−0.049 (−0.435, 0.337)	0.195	−0.253	NO (0.801)	2.20
Sex (M = 0; F = 1)	−1.223 (−3.881, 1.435)	1.341	−0.912	NO (0.364)	1.04
MVPA (mins/week)	0.008 (0.001, 0.014)	0.003	2.308	YES (0.023)	10.75
Constant	14.970 (5.881, 24.058)	4.585	3.265	YES (0.001)	
Up cvBRS					
Muscle Strengthening Frequency (days/week)	2.452 (1.633, 3.271)	0.413	5.932	YES (<0.001)	68.01^a^
Age (years)	−0.102 (−0.202, −0.003)	0.050	−2.035	YES (0.044)	15.89
BMI (kg/m^2^)	0.107 (−0.408, 0.623)	0.260	0.413	NO (0.681)	0.47
Sex (M = 0; F = 1)	−3.156 (−6.708, 0.397)	1.792	−1.761	NO (0.081)	4.99
MVPA (mins/week)	0.011 (0.002, 0.019)	0.004	2.435	YES (0.017)	10.54
Constant	7.834 (−4.313, 19.981)	6.128	1.919	NO (0.058)	
Down cvBRS					
Muscle Strengthening Frequency (days/week)	1.869 (1.237, 2.501)	0.319	5.860	YES (<0.001)	50.56^a^
Age (years)	−0.187 (−0.264, −0.110)	0.039	−4.816	YES (<0.001)	38.40^a^
BMI (kg/m^2^)	−0.092 (−0.490, 0.306)	0.201	−0.457	NO (0.649)	3.40
Sex (M = 0; F = 1)	0.551 (−2.191, 3.292)	1.383	0.398	NO (0.691)	0.39
MVPA (mins/week)	0.005 (−0.001, 0.012)	0.003	1.574	NO (0.118)	7.25
Constant	17.099 (7.725, 26.473)	4.729	4.124	YES (<0.001)	

cvBRS, cardiovagal baroreflex sensitivity; BMI, body mass index; SE, standard error; MVPA, moderate to vigorous physical activity. Significance accepted as *p* < 0.05.

^a^ If the relative weights 95% confidence intervals did not encompass zero, then they are statistically significant. It is possible for a predictor to be independently predictive of the outcome variable in multiple regression but not be a statistically significant weight to the overall R^2^.

Muscle strengthening activity frequency was not associated with SBP (overall model: adjusted R^2^ = 0.120, *p* < 0.001, *β* = 0.32, *p* = 0.497) or DBP (overall model: adjusted R^2^ = 0.129, *p* < 0.001, *β* = −0.19, *p* = 0.681), but was positively associated with RRI (overall model: adjusted R^2^ = 0.255, *β* = 0.03, both, *p* < 0.001; raw weight 0.10; Supplementary Table S1). Relative weights confirmed that age (55%) and muscle strengthening activity frequency (35%) were both predictors of RRI. Neither muscle strengthening frequency nor meeting guidelines were associated with BEI, up-BEI, or down-BEI (all, *p* > 0.14; Supplementary Table S3).

## Discussion

This study aimed to provide clarification regarding the impact of muscle strengthening activity frequency on cvBRS. In a heterogeneous group of adults, participants who engaged in more frequent weekly muscle strengthening activities had higher cvBRS after adjusting for MVPA, age, sex, and BMI, with muscle strengthening frequency exhibiting the greatest relative importance. This study highlights the benefits of regular muscle strengthening activities and meeting the associated guidelines on vagally-mediated blood pressure regulation. This study also provides a novel perspective on how more frequent bouts of muscle strengthening activity may be efficacious for beat-by-beat blood pressure control and helps clarify conflicting reports on the impact of muscle strengthening exercise on cvBRS (19, 20, 22).

The benefits of exercise on overall- and cardiovascular-specific health markers have been well established (32–36). The current resistance/muscle strengthening exercise literature on cardiovagal baroreflex function is limited and provides conflicting results (19, 20, 22). Our observations that more frequent muscle strengthening exercise sessions was associated with better cvBRS is consistent with previous findings implementing a lower body resistance training program (4–5 sets of 6–10 repetitions, combination of sets and repetitions depended on the week of the program participants were in, 2 days/week, 19 weeks) (37). Acutely, handgrip exercise augments cvBRS in healthy adults, whereby it was increased 10-minutes after a single bout of handgrip exercise completed for 4 sets of 2 min at 30% of maximal contraction force (29). In contrast to the current study and these submaximal handgrip and resistance training studies, opposing results have been observed in smaller samples of healthy young adults following heavy strength training (20) and cross-sectional studies of young males who frequently engaged in resistance training (22, 38). The current study has been conducted in a large sample including males and females of a variety of ages. The current study also focused on muscle strengthening activities specifically to align with (inter)national guidelines that may include resistance training. However, it is quite possible that these activities are completed at a much lower intensity or volume (e.g., gardening) than those of Nakamura et al. (22), in which participants were long duration (>2 years) and high frequency (>5 days/week) resistance training, and Figueroa et al. (38) whose participants were more experienced with resistance training (4–7 years) at moderate to high intensity (≥2 days/week). In addition, different methodologies of assessing cvBRS spontaneous in the present study vs. during dynamic maneuvers [e.g., handgrip exercise (38), Valsalva's Maneuver (22), etc.] that engage several physiological processes beyond the vagal arm of the arterial baroreflex (i.e., cardiopulmonary afferents, chemoreceptor afferents, pulmonary afferents, etc.) may contribute to the divergent observations. Despite the heterogeneity regarding the impact of muscle strengthening exercises on cvBRS, our study adds to the literature by supporting the overarching notion that more frequent muscle strengthening exercise is associated with better vagally-mediated blood pressure regulation, which may partially explain the positive cardiovascular effects of strengthening exercise.

This study provides evidence that participants who met muscle strengthening guidelines had greater cvBRS. Higher cvBRS indicates that there is a greater ability of the vagal arm of the baroreflex to modify beat-by-beat SBP, which is characteristic of a lower risk of cardiovascular events and hypertension (39), and maintaining normal blood pressure (40). The physiological mechanisms underpinning our observations are unclear but may be a result of muscle strengthening activity-induced vascular or neural adaptations. An umbrella review (41) reported that low-to-moderate intensity (42, 43), but not high intensity (44), resistance training led to reduced central artery arterial stiffness. A greater compliance of barosensory-containing arteries (e.g., carotid arteries) to distend during pressure would result in an improved mechanical component of the cardiovagal baroreflex (43). As well, physical activity has been linked to greater concentrations of muscarinic receptors at the sinoatrial node (45), and structural remodelling of the neurons in the nucleus tractus solitarius of the medulla oblongata, a brain region that receives neural signals from baroreceptors involved in the cardiovagal baroreflex arc (46). Whether these neural changes are stimulated by frequent muscle strengthening activities is unclear but worthy of future study.

This present study is strengthened by its investigation into a large sample of male and female participants across a wide age range. This study is also strengthened in its assessment of habitual muscle strengthening activities in consideration of (inter)national guidelines, which coincides with public health messaging. We did not focus on direct measures of muscle strength as the objective of this study was to determine how muscle strengthening frequencies and meeting physical activity guidelines, not muscle strength *per se*, was associated with cvBRS. However, this study does not come without certain limitations. This study is cross-sectional in nature, limiting the ability to establish causal relationships between muscle strengthening and cvBRS. Future studies should consider following a longitudinal approach to confirm causality by integrating muscle strengthening training and examining its impact on cvBRS. Another limitation is the use of self-report questionnaires to gather muscle strengthening frequency data from participants in which the validity of resistance training questionnaire is not well-established (e.g., number of muscle strengthening sessions reported vs. an objective criterion) and they do not specify the mode of muscle strengthening that participants engaged in. Our study was limited to the data collected based on the specific wording of the question and were unable to collect data on specific exercise type, intensity, or history of exercise. Additionally, self-report measures may introduce over- or underestimation of frequencies. Future studies should consider using objective measures of measuring muscle strengthening frequency and collecting specific data on exercise type and intensity. Interventional studies could also consider implementing groups who complete different types of exercise to determine how this influences the results we have reported. An inability to account for diet and sex hormones in the analysis can also be considered a limitation, as a healthier diet and higher levels of estrogen may augment cvBRS, although neither BMI nor sex were associated with cvBRS in our study. Nevertheless, future studies should control for diet and hormones during testing sessions if possible. Additionally, it is important to note that we were unable to replicate previous findings of a positive relationship between objectively measured MVPA and cvBRS, which was reported using a subsample of the population from this study, providing further evidence to support the discrepancy between objective vs. subjective assessment of MVPA. This study is not mechanistic in approach and does not include measures that might explain changes in cvBRS such as arterial distensibility (47), central artery stiffness (41), neural changes to the concentration of muscarinic receptors on the sinoatrial node (45), the anatomy of the neurons in the nucleus tractus solitarius (46), or vagus nerve activity using microneurography (48). Future work should focus on measuring the neural adaptations and arterial stiffness to clarify the effects of exercise on these structures involved in autonomic function to improve the understanding of the mechanisms underlying this relationship.

This study provides evidence that higher frequencies of muscle strengthening activities are associated with improved cvBRS and meeting the current physical activity guidelines recommending muscle strengthening activities at least twice per week was independently associated with better vagally-mediated blood pressure regulation. Overall, this study highlights the cardiovascular benefits of individuals engaging in muscle strengthening activities and provides evidence for some of the benefits of achieving public health recommendations for muscle strengthening activities on beat-by-beat blood pressure regulation.

## Data Availability

The raw data supporting the conclusions of this article will be made available by the authors, upon reasonable request.
