# Supplementary material for: Meeting international self-report muscle strengthening guidelines is associated with better cardiovagal baroreflex sensitivity in adults
Source: Front Sports Act Living. 2024 Dec 11;6:1509784. doi: 10.3389/fspor.2024.1509784 (PMC11668578; doi:10.3389/fspor.2024.1509784)
Supplement: Supplementary file 1 [file Table1.docx]

**Supplemental Figure 1.** Multiple regression of resistance training frequency and spontaneously measured cardiovagal baroreflex sensitivity up sequences. Participants (n = 114, 62 females) are grouped by age and sex, with triangles representing males, circles representing females, grey representing those 55 years or younger, and white representing those over 55 years. The multiple regressions included moderate-to-vigorous physical activity, age, sex, and body mass index as covariates.
