# Supplementary material for: Meeting international self-report muscle strengthening guidelines is associated with better cardiovagal baroreflex sensitivity in adults
Source: Front Sports Act Living. 2024 Dec 11;6:1509784. doi: 10.3389/fspor.2024.1509784 (PMC11668578; doi:10.3389/fspor.2024.1509784)
Supplement: Supplementary file 2 [file Table2.docx]

**Supplemental Table 2** Multiple regression analyses examining cardiovagal baroreflex sensitivity measures and their relationship to meeting muscle strengthening guidelines of at least twice per week.

| Variable | Unstandardized $\beta$  (95% CI) | SE | *t*-value | Significant predictor  (*p-*value) | Relative Weight (% of 100%) |
| --- | --- | --- | --- | --- | --- |
| ***Overall cvBRS*** | |  |  |  |  |
| Meeting Muscle Strengthening Guidelines (≥2 days/week) | 7.233  (4.282, 10.185) | 1.489 | 4.858 | **YES (<0.001)** | 49.37* |
| Age (years) | -0.150  (-0.232, -0.068) | 0.041 | -3.628 | **YES (<0.001)** | 32.61* |
| BMI (kg/m^2^) | 0.004  (-0.417, 0.425) | 0.212 | 0.020 | NO (0.984) | 2.36 |
| Sex (M=0; F=1) | -1.841  (-4.794, 1.111) | 1.489 | -1.236 | NO (0.219) | 2.34 |
| MVPA (mins/week) | 0.007  (-22.300E-5, 0.014) | 0.004 | 1.921 | NO (0.057) | 13.32 |
| Constant | 15.041  (5.081, 25.001) | 5.025 | 2.993 | **YES (<0.003)** |  |
| ***Up cvBRS*** |  |  |  |  |  |
| Meeting Muscle Strengthening Guidelines (≥2 days/week) | 7.901  (4.027, 11.774) | 1.954 | 4.043 | **YES (<0.001)** | 54.68* |
| Age (years) | -0.092  (-0.200, 0.015) | 0.054 | -1.706 | NO (0.091) | 13.99 |
| BMI (kg/m^2^) | 0.168  (-0.384, 0.721) | 0.279 | 0.604 | NO (0.547) | 0.57 |
| Sex (M=0; F=1) | -3.800  (-7.675, 0.075) | 1.955 | -1.944 | NO (0.054) | 8.99 |
| MVPA (mins/week) | 0.010  (0.001, 0.020) | 0.005 | -1.944 | **YES (0.036)** | 21.78 |
| Constant | 8.025  (-5.046, 21.097) | 6.594 | 1.217 | NO (0.226) |  |
| ***Down cvBRS*** |  |  |  |  |  |
| Meeting Muscle Strengthening Guidelines (≥2 days/week) | 6.160  (3.186, 9.134) | 1.500 | 4.105 | **YES (<0.001)** | 41.00* |
| Age (years) | -0.179  (-0.261, -0.096) | 0.042 | -4.301 | **YES (<0.001)** | 46.23* |
| BMI (kg/m^2^) | -0.046  (-0.470, 0.379) | 0.214 | -0.213 | NO (0.832) | 3.68 |
| Sex (M=0; F=1) | 0.033  (-2.943, 3.008) | 1.501 | 0.022 | NO (0.983) | 0.55 |
| MVPA (mins/week) | 0.005  (-0.002, 0.012) | 0.004 | 1.321 | NO (0.189) | 8.55 |
| Constant | 17.190  (7.154, 27.227) | 5.063 | 3.395 | **YES (<0.001)** |  |
|  |  |  |  |  |  |

cvBRS, cardiovagal baroreflex sensitivity; BMI, body mass index; SE, standard error; MVPA, moderate to vigorous physical activity. Significance accepted as *p* < 0.05. *If the relative weights 95% confidence intervals did not encompass zero, then they are statistically significant. It is possible for a predictor to be independently predictive of the outcome variable in multiple regression but not be a statistically significant weight to the overall R^2^.
