# Supplementary material for: Meeting international self-report muscle strengthening guidelines is associated with better cardiovagal baroreflex sensitivity in adults
Source: Front Sports Act Living. 2024 Dec 11;6:1509784. doi: 10.3389/fspor.2024.1509784 (PMC11668578; doi:10.3389/fspor.2024.1509784)
Supplement: Supplementary file 3 [file Table3.docx]

**Supplemental Table 1.** Multiple regression analyses examining effect of muscle strengthening frequency on systolic blood pressure and R-R intervals.

| Variable | Unstandardized $\beta$  (95% CI) | SE | *t*-value | Significant predictor  (*p-*value) | Relative Weight (% of 100%) |
| --- | --- | --- | --- | --- | --- |
| ***SBP Model*** |  |  |  |  |  |
| Muscle Strengthening Frequency (days/week) | 0.315  (-0.601, 1.232) | 0.462 | 0.682 | NO (0.497) | 2.03 |
| Age (years) | 0.155  (0.043, 0.266) | 0.056 | 2.754 | **YES (0.007)** | 52.77* |
| BMI (kg/m^2^) | 0.670  (0.094, 1.247) | 0.291 | 2.304 | **YES (0.023)** | 40.72 |
| Sex (M=0; F=1) | -2.124  (-6.098, 1.850) | 2.005 | -1.059 | NO (0.292) | 2.71 |
| MVPA (mins/week) | -0.001  (-0.011, 0.009) | 0.005 | -2.530 | NO (0.801) | 1.77 |
| Constant | 95.3  (81.7, 108.9) | 6.855 | 13.906 | **YES (<0.001)** |  |
| ***RRI Model*** |  |  |  |  |  |
| Muscle Strengthening Frequency (days/week) | 0.028  (0.014, 0.042) | 0.007 | 3.895 | **YES (<0.001)** | 34.95 |
| Age (years) | 0.004  (0.003, 0.006) | 0.001 | 5.039 | **YES (<0.001)** | 54.75* |
| BMI (kg/m^2^) | -0.005  (-0.014, 0.004) | 0.005 | -1.195 | NO (0.235) | 1.86 |
| Sex (M=0; F=1) | 0.040  (-0.022, 0.101) | 0.031 | 1.271 | NO (0.206) | 5.42 |
| MVPA (mins/week) | 9.532E-5  (5.600E-5, 24.700E-5) | 7.600E-5 | 1.250 | NO (0.214) | 3.03 |
| Constant | 0.874  (0.664, 1.085) | 0.106 | 8.227 | **YES (<0.001)** |  |
|  |  |  |  |  |  |

SBP, systolic blood pressure (mmHg); BMI, body mass index; RRI, R-R interval (ms); SE, standard error; MVPA, moderate to vigorous physical activity. Significance accepted as *p* < 0.05. *If the relative weights 95% confidence intervals did not encompass zero, then they are statistically significant. It is possible for a predictor to be independently predictive of the outcome variable in multiple regression but not be a statistically significant weight to the overall R^2^.
