# Supplementary material for: Meeting international self-report muscle strengthening guidelines is associated with better cardiovagal baroreflex sensitivity in adults
Source: Front Sports Act Living. 2024 Dec 11;6:1509784. doi: 10.3389/fspor.2024.1509784 (PMC11668578; doi:10.3389/fspor.2024.1509784)
Supplement: Supplementary file 5 [file Table5.docx]

**Supplemental Figure 3** Comparing the spontaneously measured cardiovagal baroreflex sensitivity up sequences of those who met muscle strengthening guidelines to those who did not. Participants (n = 114, 62 females) are grouped by sex and age with triangles representing males, circles representing females, grey representing those 55 years or younger, and white representing those over 55 years. Mean and standard deviation of each group, those that did not meet guidelines and those that did are represented by the large black circles. The multiple regression included moderate-to-vigorous physical activity, age, sex, and body mass index as covariates.
